# The child transgender patient in primary care: practical advice for a 10-minute consultation

**DOI:** 10.3399/bjgpopen17X101169

**Published:** 2017-10-04

**Authors:** Charlotte Cliffe, Miriam Hillyard, Albert Joseph, Azeem Majeed

**Affiliations:** 1 Honorary Clinical Research Fellow, Department of Primary Care and Public Health, Imperial College London, London, UK; 2 Foundation Doctor, North West Thames Foundation School, Imperial College Healthcare NHS Trust, London, UK; 3 Honorary Clinical Research Fellow, Department of Primary Care and Public Health, Imperial College London, London, UK; 4 Professor of Primary Care, Department of Primary Care and Public Health, Imperial College London, London, UK

**Keywords:** transgender, gender identity, children transgender, adolescent transgender

## Background

Children and adults with gender identity concerns are increasingly presenting for treatment, with referrals to specialist clinics rapidly rising. The percentage of children with gender identity disorder that self-harm or attempt suicide is estimated at 50%, so it is essential that it is recognised early and managed appropriately.^1^

Gender identity disorder of childhood usually manifests before puberty. The child typically experiences distress resulting from an incongruence between their current gender identity (sense of themselves as ‘male’, ‘female’, or otherwise), and their gender assigned at birth. Behaviour and activities of the child may stereotypically be associated with that of the opposite gender and the child may be preoccupied with wanting to change their name and gender pronoun (‘she’, ‘he’). Depending on the age, they may also have a strong desire to acquire secondary sexual characteristics of the opposite gender. This may cause significant distress and can impact their performance and experiences at school or at home. Six months of persistent gender non-conforming behaviour has been proposed as an indicator that this is more than ‘a phase’, which is common and not necessarily pathological for many individuals in childhood.

## Example case

Tom is 13 years old and has come with his mother to see you. She tells you he becomes distressed when people address him as Tom, and asks to be called Kelly. He hates wearing boys’ clothes, and most of his friends at school are girls, leading to teasing about him being ‘gay’. Recently, his mother found a skirt in his room, which he eventually admitted to having stolen from a friend’s house. He has asked his mother if he can ‘become a girl’, and seems so preoccupied with this idea that he is struggling to complete his school homework. Today Tom’s mother wants to know what to do.

## What needs to be ascertained?

### Gather a history of gender identity concerns

Find out how long this has been going on for, if there were any particular triggers, and ask for more detail on the specific behaviours that are concerning the mother or other family members. Ask Tom, in his own words, what ‘becoming a girl’ means for him. Establish both the mother's and Tom’s expectations from the consultation and what their understanding of gender identity is. Does the child have a normal developmental history, or any other comorbid diagnoses (such as learning disability, autism, or atypical anatomy)?

### Social history

Determine if Tom has siblings, other biological or step-parents, close family members, and gently ascertain whether they are supportive. Does the family already have any contact with social services? Is the child particularly vulnerable (for example, has pre-existing safeguarding concerns, or is a looked-after child)? Remember patients may come from families or cultural contexts where gender roles are rigidly, or even violently, enforced.

### Screen for depression and self-harming or suicidal thoughts

Rates are extremely high in children with gender identity concerns. If Tom is distressed, it is important to find out the level of distress, whether he has thoughts of self-harm or suicide, and whether the distress is specifically about his gender/body or if there is something else going on (for example, bullying or difficult family relationships). Are there any other risk behaviours such as drug or alcohol abuse, or unprotected sexual activity?

### Are the school supportive?

Do they know about what is happening, and have they appointed anyone in the school as an advocate? Support in the school environment is crucial to preventing long-term mental health problems. Most schools will have an equality policy that protects transgender children from harassment and discrimination, but not all schools will be equipped to enact this.^2^

### Does Tom have any symptoms or signs of puberty?

This affects the urgency of the referral to the specialist services. If puberty has started, both a referral to the specialist clinic and the local child and adolescent mental health services (CAMHS) needs to be **urgent.^3^** Pubertal changes (for example, menarche or voice breaking) may sharply increase the level of gender-related distress a child is experiencing.

## Actions advised

- Depending on the level of distress, a referral to both the local CAMHS service and specialist gender identity development service (GIDS) should be urgent. If Tom is suicidal, self-harming, or extremely distressed, an **urgent** referral is warranted. If the child is not suicidal, then a routine referral to CAMHS is warranted as ongoing psychological support will be required.
- Assess whether Tom is ‘Gillick competent’. There is a question about consent in this age group, particularly about potentially complex treatment decisions such as gender reassignment. For the majority of children, gender identity concerns will resolve before adulthood (roughly 75%).^4^ However, when the distress does not resolve, the benefits of treatment before puberty might be enormous, and would avoid the need for later surgical procedures (with associated risks) aimed at reversing secondary sexual characteristics. Due to the difficult nature of these questions, cases should be considered on an individual basis. Involvement of an ethics committee is recommended for particularly difficult cases.^5^
- Determine the attitudes of the parents or guardians regarding referral to specialist gender services.
- Refer to GIDS if appropriate; where further decisions about management and intervention can be made (see Box 1). In general, GIDS will only accept referrals for patients with identified risk behaviours if there is already local CAMHS support or a CAMHS referral.
- Discuss the importance of the school and its ability to provide an advocate, as support in the school environment is key.^2^–^3^

**Box 1.** Hormone therapy for children and adolescents with gender identity disorder

Hormonal therapies are only prescribed in specialist clinics with input from a paediatric endocrinologist. In children who have not started puberty, hormones are not normally advised. In those who have started puberty (Tanner stage 2 and above) a gonadotrophin hormone receptor blocker can be started to halt the development of secondary sexual characteristics. This is reversible, and allows time for the child or adolescent to decide preferred gender at an older age, at which point decisions about starting oestrogens or testosterones can be made on a more informed basis. Surgical gender reassignment procedures are not usually recommended before the age of 18 years.^1^

## Long-term follow-up

Specialists will guide this. However, GPs may play a role in performing baseline and monitoring bloods (under specialist advice). Ongoing psychological input may be required; the mental health specialists, within CAMHS, usually direct this. Providing support and acting in a role of advocacy for the child and their family remains a key role for the GP, throughout a process which is likely to be challenging for them all.^6^
